# Semi-supervised Ensemble Learning for Automatic Interpretation of Lung Ultrasound Videos

**DOI:** 10.1007/s10278-024-01344-y

**Published:** 2024-12-13

**Authors:** Bárbara Malainho, João Freitas, Catarina Rodrigues, Ana Claudia Tonelli, André Santanchè, Marco A. Carvalho-Filho, Jaime C. Fonseca, Sandro Queirós

**Affiliations:** 1 Life and Health Sciences Research Institute, School of Medicine, University of Minho, Braga, Portugal; 2 ICVS/3B’s - PT Government Associate Laboratory, Braga/Guimarães, Portugal; 3 Algoritmi Center, School of Engineering, University of Minho, Guimarães, Portugal; 4 Department of Internal Medicine, Hospital Clínicas de Porto Alegre, Porto Alegre, Brazil; 5 Institute of Computing, University of Campinas, São Paulo, Brazil; 6 Wenckebach Institute, Research program LEARN, University Medical Center Groningen, University of Groningen, Groningen, The Netherlands

**Keywords:** Lung ultrasonography, Video analysis, Deep learning, Semi-supervised learning

## Abstract

Point-of-care ultrasound (POCUS) stands as a safe, portable, and cost-effective imaging modality for swift bedside patient examinations. Specifically, lung ultrasonography (LUS) has proven useful in evaluating both acute and chronic pulmonary conditions. Despite its clinical value, automatic LUS interpretation remains relatively unexplored, particularly in multi-label contexts. This work proposes a novel deep learning (DL) framework tailored for interpreting lung POCUS videos, whose outputs are the finding(s) present in these videos (such as A-lines, B-lines, or consolidations). The pipeline, based on a residual (2+1)D architecture, initiates with a pre-processing routine for video masking and standardisation, and employs a semi-supervised approach to harness available unlabeled data. Additionally, we introduce an ensemble modeling strategy that aggregates outputs from models trained to predict distinct label sets, thereby leveraging the hierarchical nature of LUS findings. The proposed framework and its building blocks were evaluated through extensive experiments with both multi-class and multi-label models, highlighting its versatility. In a held-out test set, the categorical proposal, suited for expedite triage, achieved an average F1-score of 92.4%, while the multi-label proposal, helpful for patient management and referral, achieved an average F1-score of 70.5% across five relevant LUS findings. Overall, the semi-supervised methodology contributed significantly to improved performance, while the proposed hierarchy-aware ensemble provided moderate additional gains.

**Supplementary Information:**

The online version contains supplementary material available at 10.1007/s10278-024-01344-y.

## Introduction

The respiratory system, with its warm and humid environment, provides an ideal breeding ground for airborne pathogens, rendering it highly vulnerable to disease. Respiratory diseases constitute a significant global health worldwide, accounting for >10% of all disability-adjusted life years and for 5 of the top 30 causes of mortality [1, 2]. The lungs, as the internal organ most exposed to inhaled pathogens, are particularly susceptible to infections and injuries.

Transthoracic ultrasound is a well-established yet underutilised imaging modality for lung assessment. Offering safety, affordability, mobility, and real-time evaluation capabilities, this technique enables visualisation of pleural and chest wall abnormalities and serves as a valuable aid for minimally invasive procedures like biopsies. Point-of-care ultrasound (POCUS) concerns a portable ultrasound system designed for bedside patient assessments, especially in emergency settings. Distinguished from conventional ultrasound by its focus on swift yet reliable evaluations, POCUS plays a pivotal role in timely medical interventions [3, 4].

Interpreting lung ultrasound (LUS) poses unique challenges due to the limited wave propagation in pulmonary tissues, necessitating a keen understanding of ultrasound artifacts. These challenges underscore the importance of developing automated strategies to assist medical practitioners in identifying relevant findings in LUS, particularly in the POCUS setting, where videos often have lower image quality. Such strategies hold promise for enhancing diagnostic accuracy and improving patient outcomes.

Since LUS is not the standard imaging modality for lung assessment, fewer studies have traditionally been conducted on automated systems for LUS compared to computed tomography or chest radiography [5, 6]. Notwithstanding, recent years have witnessed a growing interest in this field, namely in the context of the SARS-CoV-2 lung infection [7–13]. Existing literature predominantly showcases three types of deep learning (DL) approaches for LUS analysis: (1) single-frame classification [11–15], which overlooks the dynamic nature of acquisitions; (2) frame-based video classification, where each frame is analyzed independently and then aggregated into a video-level prediction [8–10, 16]; and (3) video-based classification, where a video (or a segment of it) is directly fed to the network [13, 17–19]. The ability to capture the temporal dynamics of lung movement — absent in the first two approaches — is critical for detecting certain pathological findings. Moreover, most of the current research in this field targets the prediction of specific single-label diagnoses or disease severity, thereby neglecting the complexity of multi-label scenarios where multiple findings may coexist. Since effective diagnosis often hinges on assessing the presence or absence of individual findings in relation to external clinical factors, these methods fail to accurately reflect the complexity of LUS interpretation in clinical practice. However, predicting pathological findings in lung POCUS videos poses several challenges. On the one hand, it requires capturing the subtleties of each finding, but also accounting for the potential coexistence of multiple ones within a single examination. Additionally, developing such automated systems requires extensive and meticulous annotation efforts.

Recent works have made notable progress in addressing the scarcity of annotated data through semi-supervised approaches. Li et al. [20] introduced a detector-based video classification approach that leverages temporal context, thereby improving the detection accuracy of consolidations and pleural effusions by capturing critical dynamic aspects in LUS videos. Similarly, Ouyang et al. [21] proposed a weakly semi-supervised detection approach, based on a teacher-student training strategy, which enhances the identification of consolidations in LUS videos by effectively utilising limited annotated data. While these works underscore the potential of semi-supervision to mitigate the challenges posed by scarce annotations, none address the simultaneous identification of multiple coexisting findings — an aspect crucial to LUS practice.

With this in mind, our work proposes a novel framework for the computer-assisted interpretation of lung POCUS videos, specifically designed to address the intrincacies of multi-label LUS interpretation. Our framework leverages a semi-supervised DL-based classification architecture and a novel ensemble modeling strategy that exploits the inherent hierarchy of LUS interpretation to automatically detect significant findings, notably working for both multi-class (coarse video categorisation) and multi-label (detection of multiple findings) scenarios.

## Dataset

For the sake of readability and recognising the importance of the available annotations and respective label hierarchy in the proposed methodology, we first detail the in-house dataset curated and annotated specifically for this study. It comprises lung POCUS videos obtained from the Department of Internal Medicine at *Hospital de Clínicas de Porto Alegre* (HCAP), Brazil, from 2020 to 2022. Ethical approval for retrospective data collection was obtained from the Ethics Committee for Research (ECR) in Life and Health Sciences of the University of Minho (CEICVS 039/202) and the ECR of HCAP (5.334.879). Acquisitions adhered to a 12-field protocol, involving the examination of each lung and respective pleura by dividing it into superior and inferior sections, each assessed anteriorly, posteriorly, and laterally.

Ultrasound scans were captured by multiple clinicians using the SonoSite M-Turbo^®^ portable ultrasound system (SonoSite, Inc, USA) with a 2-5 MHz convex probe. Data was collected from 990 patients treated at the department, encompassing individuals both with and without symptoms, providing a diverse representation of the general population. Videos exhibit variability in frame rate, ranging from 25 to 60 frames per second, with most videos having a duration of 6 s. Videos with less than 3 s or with sub-optimal quality preventing effective interpretation were excluded. In total, the dataset comprises 8160 LUS videos of the parenchyma, stored in MP4 format. The videos have a median resolution of 488$\times$380 pixels, with sizes ranging from 278$\times$158 to 492$\times$394 pixels. All videos underwent de-identification using the *Clip Deidentifier* tool,^1^ with no additional demographic information, indication of pathology, or symptomatology recorded.

**Fig. 1:**
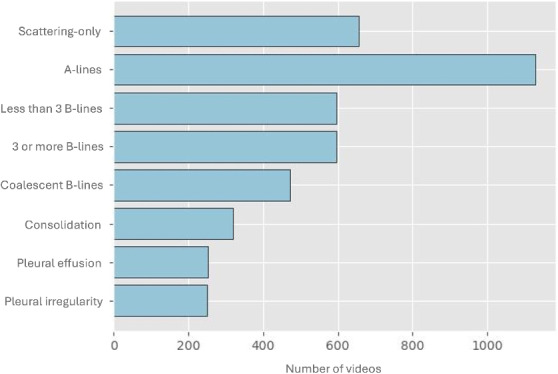
Distribution of findings within the labeled dataset subset

Two medical doctors, with 14 and 7 years of expertise in LUS, annotated the dataset at the video level. Consensus meetings were initially conducted to harmonise interpretations and definitions, considering recent guidelines [3, 4]. Then, each clinician analyzed a distinct set of videos, with no overlap between their assignments. Challenging videos were flagged for discussion, ensuring consistency and improving annotation quality. Of the 8160 videos, only 3649 were manually annotated, while 4511 remained unlabeled. Each annotated video was assigned one or more of the following findings: scattering-only (the absence of artifacts), A-lines artifact, less than 3 B-lines, 3 or more B-lines, coalescent B-lines, consolidation, and pleural effusion. Figure  1 illustrates the frequency distribution of these labels within the annotated subset.

**Fig. 2:**
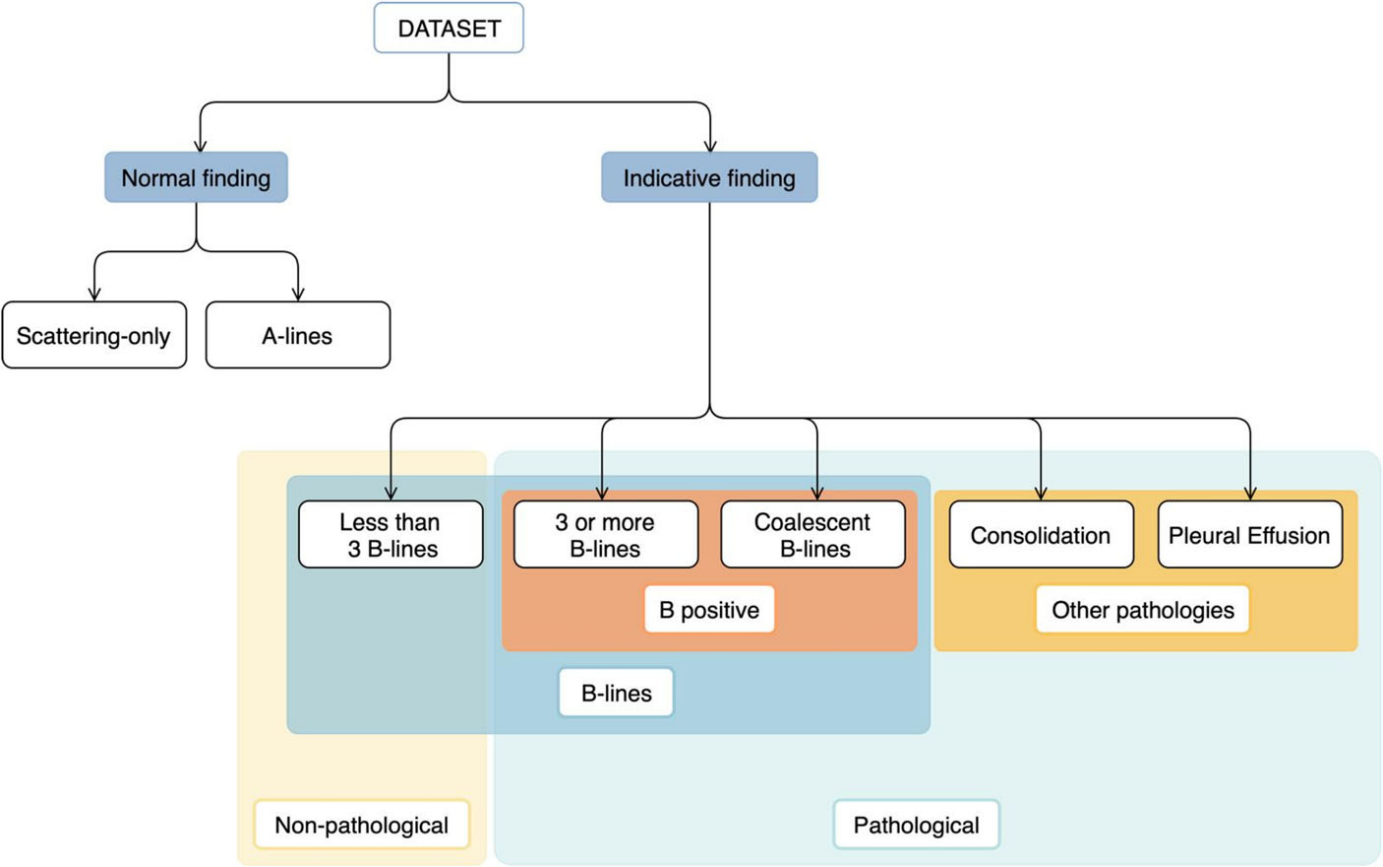
Dataset annotation categorisation

These labels can be grouped hierarchically, as depicted in Fig. 2. Findings are categorised into those indicative of normal lung function (scattering-only or A-lines) and those suggestive of underlying pathology, with the two groups being mutually exclusive. Within the latter group, there are those that, alone, are insufficient to conclude on the presence of a pathology (namely the presence of less than 3 B-lines) and those that are proof of such. Two sub-categories are found in the latter, namely the positive B-line pattern, which includes the presence of 3 or more B-lines or coalescent B-lines, and other significant pathologies, such as consolidation and pleural effusion. Among indicative findings, B-line-related labels are mutually exclusive, but they can coexist with those from the “Other pathologies” sub-category. Of note, certain findings are more subjective to interpret. Counting B-lines, for instance, can be ambiguous, as small differences can alter classification. One or two B-lines indicate a B-negative pattern, while their absence suggests a normal finding (scattering-only or A-lines). Three B-lines classify as positive, but the distinction between “$\ge$3 B-lines” and “coalescent B-lines” can also be subtle. Similarly, “consolidation” and “pleural effusion” can be misleading depending on their size and severity. This inter-observer variability, despite efforts for consensus, highlights the dataset’s susceptibility to noisy labels.

The above hierarchy and categorization will be followed throughout this work. For clarity, we further define high-level labels as categories in the hierarchy that encompass sub-categories (low-level labels). For example, a high-level label might be a broader category like “other pathologies”, while the low-level labels within it would be “consolidation” and “pleural effusion”. It is important to note that the classification of a label as high-level or low-level can vary depending on the context or specific hierarchical level being considered.

**Fig. 3:**
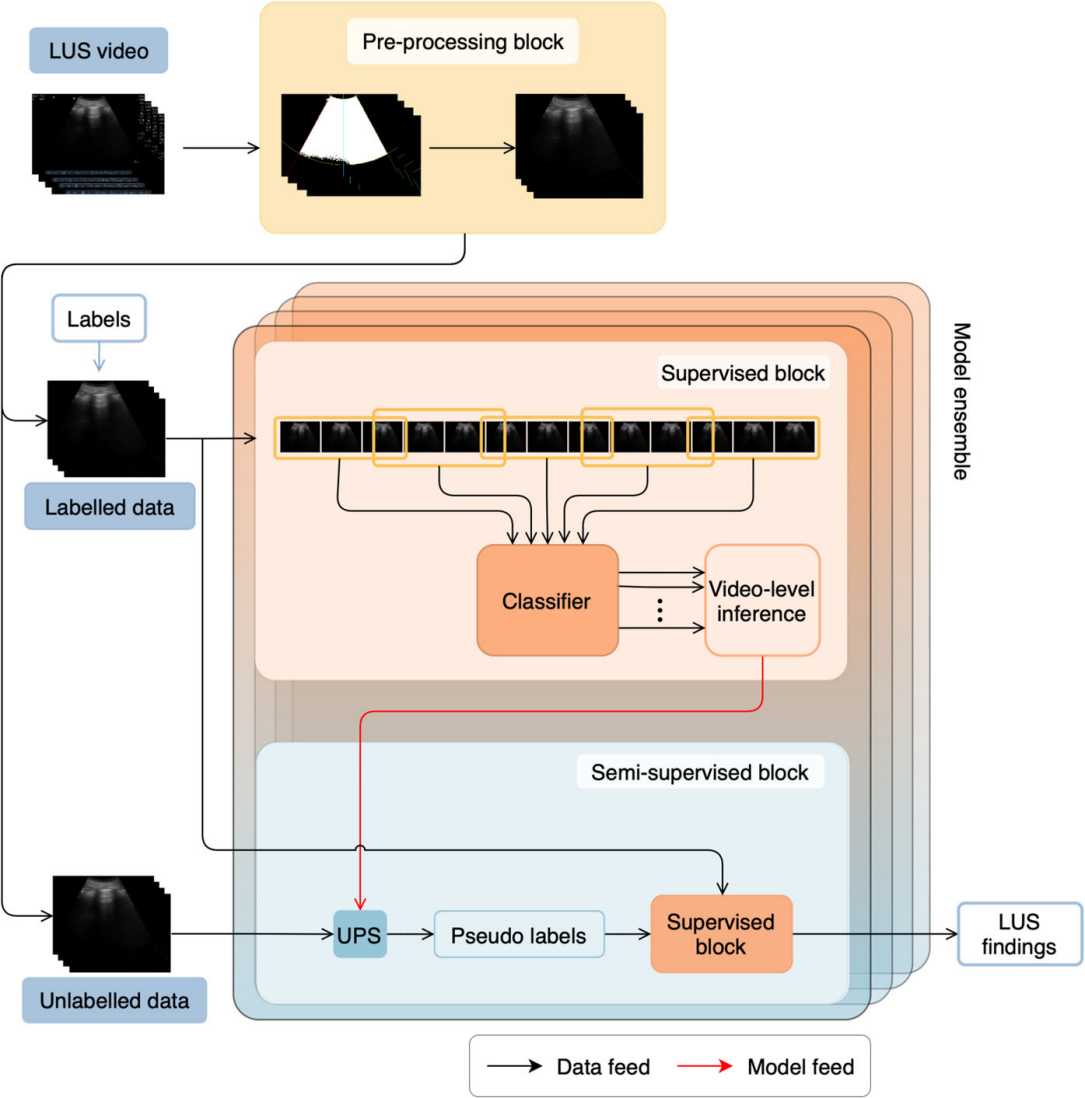
General overview of the proposed method for LUS video interpretation

## Methods

This work proposes a flexible DL-based framework for the interpretation of lung POCUS videos (Fig. 3), which rests on four core blocks: (1) a pre-processing block; (2) a supervised learning block; (3) a semi-supervised learning (SSL) block; and (4) a model ensemble block.

In brief, the pre-processing block (Sect. “Pre-processing Block”) standardises the input, masks out any information outside the field-of-view (FOV), and splits the video into multiple overlapping clips (each assumed to share the full-video labels). The supervised learning block (Sect. “Supervised Block”) utilises the available labeled data to train a 3D convolutional neural network (CNN) while employing data augmentation (Sect. “Data Augmentation”) and label smoothing regularisation. The trained classifier provides per-clip outputs that are then aggregated into a video-level prediction (Sect. “Video-Level Inference Routine”). In the SSL block (Sect. “Semi-supervised Block”), one employs the previously trained classifier to predict pseudo-labels for the unlabeled dataset and select those with high-confidence and low-uncertainty using the Uncertainty-aware Pseudo-label Selection (UPS) method [22]. Leveraging both labeled and pseudo-labeled samples, the proposed network is trained once more from scratch. The classifier’s performance is further boosted through ensemble modeling (Sect. “Ensemble Modeling”) using a novel strategy that leverages the hierarchy inherent to LUS interpretation.

### Pre-processing Block

Ultrasound videos have additional information and marks surrounding the sector scan, such as the indication of depth or details about the machine’s settings. This superfluous information may negatively influence training, as the network may focus on it during its learning process. For this reason, all videos were pre-processed to mask out any information outside the FOV using a custom masking algorithm (described in supplementary material). Details concerning the scan sector are also extracted, like the probe’s virtual origin, the radii that originate the sector’s superior and inferior arcs, and its opening angle, which will be used later in the data augmentation phase.

All videos were scaled to 128$\times$128 pixels by nearest neighbour interpolation (to minimise smoothing effects) with padding being used when needed. Additionally, pixel values were converted to grey-scale and scaled to the [0,1] range by dividing by 255.

### Supervised Block

Given the necessity for each clip to encompass a full respiratory cycle (typically around 4 s) to ensure adequate LUS assessment, the network’s input was set to 32 frames, with clips sampled at a rate of 8 Hz. In instances where videos contain an insufficient number of frames, empty frames are appended to the clip’s end.

We employ the R2+1D network with 18 layers, proposed in [23]. This network is a ResNet-based architecture and gets its name due to the factorisation of the 3D convolutional filters into spatial (2D) and temporal (1D) operations. The aim is that, by having an additional non-linearity between these operations, the number of non-linear rectification doubles compared to the non-factorised version, R3D, which translates into a model capable of performing more complex functions. Additionally, differently from 3D filters where the dynamics are intertwined, R2+1D provides an easier model to optimise [23]. Note however that, differently from [23], the proposed implementation does not consider an increased number of filters per convolutional layer, which reduces the number of parameters compared to R3D and, consequently, its memory usage and training time.

#### Data Augmentation

To increase the generalisation capabilities of the model, particularly considering it is a 3D network, data augmentation was utilised.

Although all ultrasound videos have the same beam direction (originating from the probe’s origin), in LUS exams, such fact produces particular findings (e.g. A-lines and B-lines) whose features are impossible to occur in any other direction than the beam one. Hence, there is a need for extra caution when choosing the augmentations to apply, since some could create unrealistic videos and reduce the clinical significance of the resulting classifier. To tackle this, one applies a few vanilla augmentations in the polar space instead of the Cartesian one. To perform these transformations, one needs the probe’s virtual origin (acting as the pole), as well as the sector scan’s opening angle (limits of the angular coordinate) and radii (limits of the radial coordinate). These values, as previously mentioned, were saved when the sector scan’s mask was created.

Specifically, the video clip’s scan sector is first converted to the polar space (with column and rows corresponding to angular and radial axes, respectively). In this space, a 1D scaling transform is applied over the radial axis, using the pole (i.e. the first row of the polar image) as origin. In Cartesian space, it corresponds to varying the image’s axial resolution by artificially modifying its depth. A rotation transformation is then applied through a translation along the angular axis. Again, the probe’s virtual origin is fixed, which in the Cartesian space represents the rocking of the probe. Still in polar coordinates, we apply a linear contrast augmentation. Only afterwards the clip is converted back into Cartesian coordinates using the original pole, opening angle, and radii, which guarantees that the content is still restricted to the scan’s FOV (and that the background intensity is kept unchanged). Finally, a horizontal flip transform is considered. These transformations were implemented using the Solt [24] package.

#### Video-Level Inference Routine

Videos differ from images in their mutability, as the presence or absence of findings can coexist in different segments of the same video. While the dataset was annotated at the video level, it is possible that the labeled finding is not present throughout the entire video. This can reduce the algorithm’s sensitivity if only one random clip is assessed. To tackle this, the inference process was altered.

Instead of extracting a single clip from the video and obtaining a single prediction, one divides the LUS video into multiple overlapping clips with equal length, starting the first clip in the first frame. To obtain a video-level prediction, an average of the resulting scores of the multiple clips is computed. Besides increasing the method’s sensitivity when compared to a one-clip prediction, it also increases its robustness when compared to a whole-video prediction (i.e. inputting the full video at the inference stage). Indeed, although the latter would allow the assessment of the full video and therefore allow the detection of any visible finding, by relying on multiple predictions, one decreases the method’s uncertainty and ultimately increase its accuracy.

**Fig. 4:**
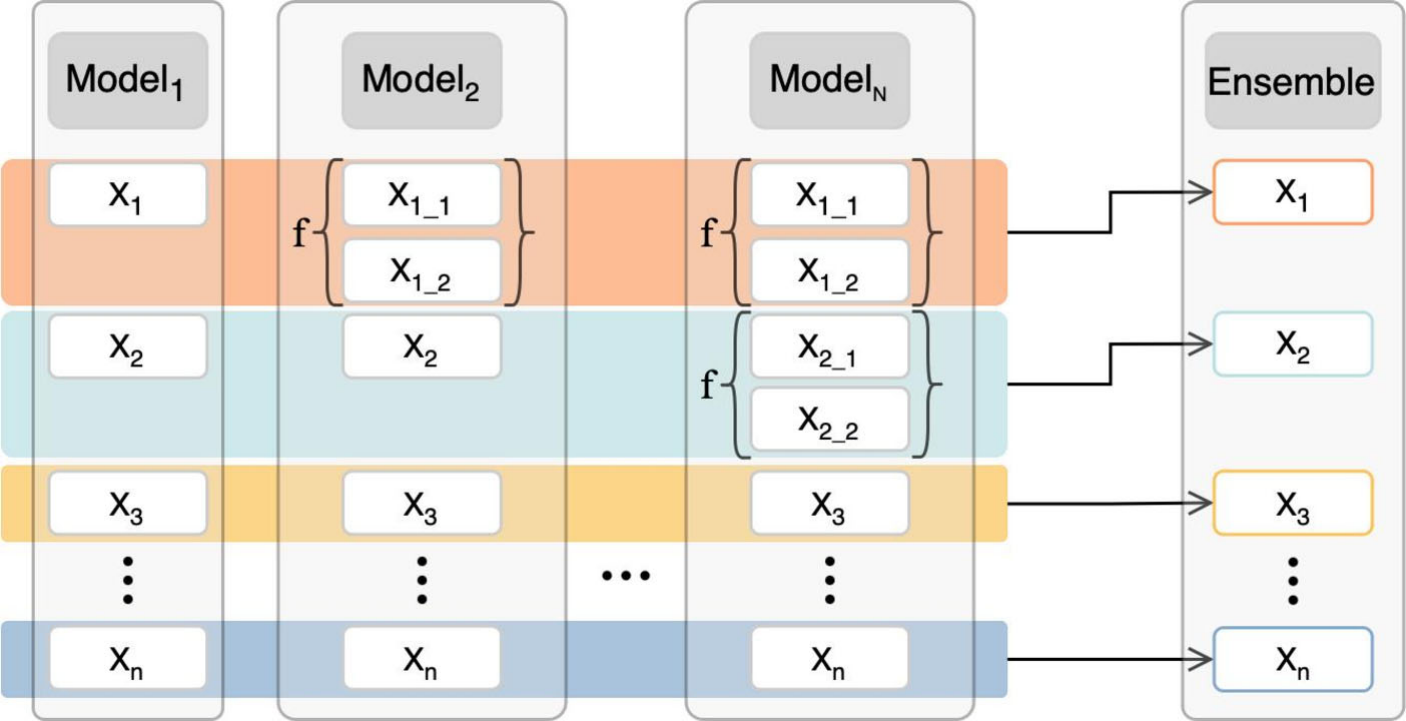
Illustration of the proposed multi-output to high-level ensemble method. The aggregation function is represent by *f* (sum/maximum function for categorical/multi-label) and the coloured blocks represent an average operation. X$_{i}$ represents the high-level feature *i*, while X$_{ij}$ denotes the low-level feature *j* associated to the high-level feature *i*

### Semi-supervised Block

For semi-supervision, one proposes to use the UPS method [22], utilising the supervised model as backbone. This is a pseudo-labeling method that, through a more precise label selection, aims to diminish the amount of noise present in the pseudo-labels. Additionally, UPS offers an approach for the use of pseudo-labeling in multi-label scenarios and performs well with video data. Note, however, that no results were originally shown for a multi-label video dataset.

The UPS method consists of three steps: pseudo-label generation, pseudo-label selection, and model training. For the generation of pseudo-labels, the video-level predictions of the supervised model are used as hard labels. However, to create more accurate labels and reduce the noise that is injected into the semi-supervised training, only high-confidence predictions are selected. This is applied in both positive and negative manner, meaning that the network can be confident that the output is of a certain class, attributing a positive label, or confident that the output does not belong to that class, giving a negative label. For this, two thresholds are defined, $\tau$_p_ the positive threshold and $\tau$_n_ the negative threshold. Besides the confidence thresholds, the prediction’s uncertainty is also integrated into the pseudo-label selection. Rizve et al. [22] showed that when labels are selected with more certainty, the calibration error is reduced. As such, two new limits are set, the uncertainty thresholds for positive and negative labels, k_p_ and k_n_, respectively. To summarise, in the multi-label setting, one has three types of pseudo-labels: positive, negative, and indeterminate. If the prediction value is higher than $\tau$_p_ and the uncertainty is lower than k_p_, the label is positive. If prediction and uncertainty are lower than $\tau$_n_ and k_n_, respectively, the label is negative. If none of the above, the label is considered indeterminate and does not contribute to the loss function. To know which labels are to be used for calculating the loss, along with the one-hot vector, a new vector is introduced, *g*. For each class *c* of each sample *i*, $g_c^{(i)}$ is either 1 (reliable) or 0 (indeterminate). In this work, label smoothing regularisation (LSR) [25] was also integrated into the UPS loss function, considering either its categorical or binary cross-entropy formulation according to the task at hand. The multi-label loss function is presented in Eq. 1, where *C* represents the number of classes, *y* the true pseudo-label vector after label smoothing, and $\hat{y}$ the prediction output. Note that, for labeled samples, all classes in *g* are set to 1.

1 $$\begin{aligned} L_{BCE} \!=\! \frac{1}{ \sum _{c} g_{c}^{(i)} } \sum _{c=1}^{C} g_{c}^{(i)} \biggr [ y_{c}^{(i)} log \Bigl ( \hat{y}_c^{(i)} \Bigl ) \!+\! \Bigl ( 1 \!-\! y_{c}^{(i)} \Bigl ) log \Bigl ( 1 \!-\! \hat{y}_c^{(i)} \Bigl ) \biggr ] \end{aligned}$$

From an implementation point-of-view, first, the supervised model is trained on the labeled dataset. Once trained, the predictions for the unlabeled dataset are calculated. Here, predictions are made to the original unlabeled clip and to nine additional augmented versions of said clip. While the prediction for the original clip is used for confidence thresholding, all ten versions are used for uncertainty estimation (computed as the standard deviation of the ten predictions). After selecting the unlabeled samples and respective pseudo-labels, these are combined with the labeled dataset and used to re-train the proposed network from scratch. After each iteration, new pseudo-labels are generated for all unlabeled samples, using the new classifier and the proposed video-level inference technique. The aim is that the pseudo-labels attributed are more curated, injecting a smaller amount of wrongly classified samples in the subsequent network training, while benefiting from the inclusion of more data.

**Table 1:** Multi-label label sets considered in the experiments

Multi-label label sets			
mlS1	mlS2	mlS3	mlS4
Scattering-only	Scattering-only	Scattering-only	Scattering-only
A-lines	A-lines	A-lines	A-lines
$<3$ B-lines	$<3$ B-lines	$<3$ B-lines	$<3$ B-lines
**B positive**	**B positive**	$\ge$3 B-lines	$\ge$3 B-lines
		Coalescent B-lines	Coalescent B-lines
**Other pathologies**	Consolidation	**Other pathologies**	Consolidation
	Pleural effusion		Pleural effusion

High-level labels are presented in bold for clarity

**Table 2:** Comparison between supervised and semi-supervised learning

	$\tau$_p_	$\tau$_n_	k_p_	k_n_	AP	F1-score	
						Macro	Micro
Supervised learning	-	-	-	-	**0**.**6812**	0.6311*	0.6780*
Pseudo-labeling	0.5	0.5	-	-	0.6580*	0.6542	0.6918
	0.5	0.05	-	-	0.6595*	0.6529	0.6892*
UPS [22]	0.5	0.05	0.05	0.005	0.6786	**0**.**6638**	**0**.**7001**

*p < 0.05, in a multiple comparison Finner post-hoc test against UPS

### Ensemble Modeling

The integration of domain knowledge in DL approaches received considerable acclaim [8, 12, 17, 26]. By leveraging the known characteristics of the data, one may adapt a framework and tailor it accordingly, often leading to performance gains. With that in mind, a novel ensemble modeling technique is here proposed, taking advantage of the hierarchy of LUS findings (Fig. 2).

The proposal consists of an ensemble of models trained to predict a different output label set, according to the dataset hierarchy present in Fig. 2. The multiple models’ outputs comprise both low- and high-level labels. All models must comprehend the same label categories. In other words, a model may either have the high-level category as a label or all of the low-level labels of said category. For example, a model can either have the “other pathologies” label, or both “consolidation” and “pleural effusion” labels. It cannot, however, have labels from a category not included in all models.

The intuition is that the classification of high-level nodes can be improved by considering information from their low-level counterparts. The reason for prioritising high-level nodes is that they are often of high clinical relevance, namely for patient screening and management, but have higher intra-class variability, which can increase the classifier’s uncertainty and undermine its performance. By contrast, low-level labels typically exhibit lower intra-class variability, enabling a classifier trained to predict them to focus on more subtle features and achieve superior performance. As such, models trained on low-level labels can be leveraged to enhance models with high-level labels, making use of knowledge that would otherwise be difficult to capture using a high-level model alone.

The proposed ensemble is obtained by combining the predictions of low- and high-level labels from the multiple models (Fig. 4). For each label, an average is computed. If the label is a low-level one (and the corresponding high-level label is not comprised in any model), the average is straightforward, considering the values inferred by all models. If the label is high-level, the process requires additional processing steps. The average prediction is derived from two sources: predictions for the high-level label from models that include it, and an aggregated score from its associated low-level labels. This aggregation varies depending on whether the problem is categorical or multi-label. In a categorical scenario, where model outputs are processed through a softmax function, the sum of all labels’ values equals 1. Consequently, the probability of a high-level label is calculated by summing the probabilities of its associated low-level labels. For the multi-label scenario, where labels are treated independently, the probability of a high-level label is determined by taking the highest predicted score among its associated low-level labels.

Note that, when the purpose is to train a classifier able to detect low-level labels only, the proposed technique comes down to a traditional ensemble modeling. In short, the same model (the one with the intended label set) is trained multiple times and the results are averaged. Independently of the scenario, the training of each one of the models is done independently.

**Table 3:** Comparison between model ensemble strategies

Label set	Ensemble type	AP	F1-score	
			Macro	Micro
mlS1	None	0.7434*	0.7027*	0.7368*
	Test-time augmentation	0.7509*	0.7111*	0.7408*
	Model repetition	0.7609	0.7181	0.7489
	Low to high-level	0.7596	**0**.**7209**	**0**.**7543**
	Multi-output to high-level (proposed)	**0**.**7694**	**0**.**7209**	0.7518
mlS4	None	0.6786*	0.6638	0.7001*
	Test-time augmentation	0.6898	0.6689	0.7041
	Model repetition (proposed)	**0**.**6994**	**0**.**6761**	**0**.**7116**

*p < 0.05, in a multiple comparison Finner post-hoc test against the proposed ensemble

**Table 4:** F1-scores of the proposal and two baselines on the test set for mlS1

Model	Macro	Micro	S	A	<3 B	B+	OP
Baseline 1	0.6611*	0.7281*	0.6612	0.8254	0.4399*	0.8446	0.546
Baseline 2	0.6730*	0.7344*	0.6748	0.8276	0.4630*	0.8435	0.5563
Proposal	**0**.**7045**	**0**.**7540**	**0**.**6925**	**0**.**8475**	**0**.**5529**	**0**.**8572**	**0**.**5725**

S: Scattering-only; A: A-lines; <3 B: Less than 3 B-lines; B+: B positive; OP: Other pathologies; *p < 0.05, in a multiple comparison Finner post-hoc test against the proposal

### Implementation Details

Categorical and binary cross-entropy were employed, respectively, as loss function in the multi-class and multi-label scenarios, with LSR applied with a factor of 0.1. Additionally, in the inference routine, the overlapping clips were extracted with a step of 0.5 s between each other.

In the semi-supervised block, $\tau$_p_, $\tau$_n_, k_p_, and k_n_ were set to 0.5, 0.05, 0.05, and 0.005, respectively.

For data augmentation, we employed scaling within ±30%, rotations up to ±10°, and a contrast gain up to 0.25. Spatial- and intensity-based transformations had a respective 50% and 15% probability of application. In the pseudo-label generation process, we utilised spatial transformations only but with reduced ranges. Specifically, scaling and rotations of up to ±7.5% and ±2.5°, respectively, were always applied, while the flip transform had a 50% probability of application.

In both supervised and semi-supervised settings, the Adam optimiser [27] was employed, running 100 epochs with a batch size of 4. The learning rate was initialised at 1$\times$10^-3^, and updated using a cosine decay learning schedule [28]. The pipeline was developed in TensorFlow, resorting to a workstation with a Nvidia RTX A6000, 64 GB of RAM, and an Intel(R) Core(TM) i9-12900F CPU.

The labeled dataset was split, at the patient-level, 80% for training and 20% for testing, with the training portion used in a 5-fold cross-validation during algorithm development. Training was performed in a mixed precision setting.

## Experiments and Results

The experiments presented herein aim to validate the performance of the proposed framework, focusing specifically on the two central aspects of our study: the semi-supervised learning module (Sect. “Semi-supervised Training”) and the novel ensemble modeling strategy (Sect. “Ensemble Modeling”). Both aspects were evaluated within the multi-label scenario due to its clinical relevance and inherent complexity. Distinct multi-label label sets (mlS) were considered for this evaluation, as summarised in Table 1.

For space sake, other ablation studies—addressing architecture selection, input size, and inference routine significance — are included in supplementary material. Briefly, these supplementary results show that our implementation of the R2+1D network outperforms other CNN models, such as R3D, C2+1D, C2D, and X3D-S, as well as the original R2+1D architecture with an increased number of channels. Furthermore, performance was shown to be stable across varying clip durations and frame rates, with the proposed multi-clip inference yielding improved results over single-clip and whole-video alternatives.

The results of Sects. “Semi-supervised Training” and “Ensemble Modeling,” along with supplementary data, represent average performance over five-fold cross-validation. In turn, Sect. “Test Set” provides the test set results, reflecting the average performance of the five trained models applied to the test subset, for both categorical and multi-label settings.

To compare models and assess significant differences, we employed the Iman-Davenport non-parametric test [29, 30]. For multiple comparisons, the post-hoc Finner test [29] was used. A significance level of 0.05 was applied to all tests.

**Table 5:** F1-scores of the proposal and two baselines on the test set for msL4

Model	Macro	Micro	S	A	<3 B	$\ge$3 B	CB	C	PE
Baseline 1	0.6070*	0.6633*	0.6662	0.8191*	0.4641*	0.6142*	0.7129*	0.5963	0.3764
Baseline 2	0.6251*	0.6760*	**0**.**6754**	0.8250*	0.4988*	0.5965*	0.7350*	**0**.**6199**	0.4249
Proposal	**0**.**6509**	**0**.**6957**	0.6702	**0**.**8433**	**0**.**5414**	**0**.**6530**	**0**.**7567**	0.6139	**0**.**4780**

S: Scattering-only; A: A-lines; <3 B: Less than 3 B-lines; $\ge$3 B: 3 or more B-lines; CB: Coalescent B-lines; C: Consolidation; PE: Pleural effusion; *p < 0.05, in a multiple comparison Finner post-hoc test against the proposal

**Table 6:** Categorical label sets considered in the experiments

Categorical label sets			
cLS1	cLS2	cLS3	cLS4
**Normal finding**	**Normal finding**	Scattering-only	Scattering-only
		A-lines	A-lines
**Indicative finding**	Non-pathological	**Indicative finding**	Non-pathological
	Pathological		Pathological

High-level labels are presented in bold for clarity

### Semi-supervised Training

The proposed framework allows the classification of any combination of high-level and low-level labels (see examples in Table 1). However, since mlS4 entails the highest number of outputs and is the most challenging task to address in the semi-supervised setting, it serves as the label set used to assess the efficacy of the proposed block.

Performance was compared against the baseline supervised setting. Additionally, to ascertain the necessity of considering uncertainty for pseudo-label selection, one also compared against the standard pseudo-labeling approach (confidence-based selection only). In the latter, classes with a prediction score above 0.5 are classified as positive (set to 1), or otherwise considered negative (set to 0). A variant with a more conservative negative confidence threshold is also included. Table 2 presents the obtained results, reporting average precision (AP), and macro and micro-averaged F1-scores. All models underwent training under identical conditions, using the inference routine outlined in Sect. “Video-Level Inference Routine”, albeit excluding ensemble modeling.

### Ensemble Modeling

To further improve the classifier’s performance, both in supervised and semi-supervised stages, we propose the use of ensemble modeling (Sect. “Ensemble Modeling”). Specifically, we introduce a novel ensemble technique based on models with distinct output label sets when targeting the classification of high-level labels (like seen for mlS1). In the case of models with low-level labels only (like mlS4), such strategy boils down to the traditional model repetition technique. Here, we compare the proposed strategy against others found in the literature for test-time improvements.

Starting with the high-level labels ensemble, our proposal involves leveraging four models (M1 to M4) trained to predict four distinct label sets (mlS1 to mlS4, respectively) and combined to predict the coarser label set (mlS1). This ensemble, termed multi-output to high-level ensemble, is compared against three alternatives: test-time augmentation (repeating inference for 9 augmented versions of the input video, following the augmentation scheme described in Sect. “Implementation Details”), model repetition ensemble (by repeating training of model M1 four times), and low-level to high-level ensemble (a variant of ours wherein the training of model M4 is repeated four times, and its low-level labels are aggregated to obtain predictions for mlS1). These results are summarised in Table 3. It is worth noting that, excluding test-time augmentation, all strategies entail the training of four models.

For the low-level labels ensemble (mlS4), the proposed strategy (that comes down to a 4-times model repetition ensemble) was compared against test-time augmentation, with the results also included in Table 3.

### Test Set

A final evaluation was conducted on a held-out test set, with results presented for both mlS1 and mlS4 and compared against two supervised baselines (Tables 4 and 5). Baseline 1 involves training the proposed R(2+1D) network using only annotated data, without additional enhancements. Baseline 2 builds on this by incorporating LSR, video-level inference, and the proposed ensemble modeling technique. The proposed method further extends baseline 2 by introducing a SSL stage.

Additionally, to demonstrate the framework’s versatility, the proposed method was applied to categorical label sets (cLS), where only mutually exclusive labels were considered (Table 6). In these experiments, LSR was not employed, as reduced label noise is expected due to mutual exclusivity. Results for both cLS1 and cLS4 are presented in Tables 7 and 8, and compared to the baselines.

**Table 7:** Performance of the proposal and two baselines on the test set for cLS1

Model	BA	MCC	AP	F1-score			
				Macro	Micro	Normal	Indicative
Baseline 1	0.9120*	0.8244*	0.9622*	0.9120*	0.9134*	0.9010*	0.9230*
Baseline 2	**0**.**9261**	**0**.**8522**	0.9678	**0**.**9261**	**0**.**9272**	**0**.**9170**	**0**.**9351**
Proposal	0.9247	0.8483	**0**.**9679**	0.9241	0.9255	0.9139	0.9343

BA: balanced accuracy; MCC: Matthews correlation coefficient; AP: average precision; *p < 0.05, in a multiple comparison Finner post-hoc text against the proposal

**Table 8:** Performance of the proposal and two baselines on the test set for cLS4

Method	BA	MCC	AP	F1-score			
				S	A	Non-path	Path
Baseline 1	0.6786*	0.6503*	0.7289*	0.6286*	0.8058*	0.4348	0.8624*
Baseline 2	0.6900	0.6657	**0**.**7459**	0.6561	0.8121	0.4264	0.8729
Proposal	**0**.**6950**	**0**.**6769**	0.7456	**0**.**6606**	**0**.**8232**	**0**.**4369**	**0**.**8762**

BA: balanced accuracy; MCC: Matthews correlation coefficient; AP: average precision; S: Scattering-only; A: A-lines; Non-path: non-pathological; Path: pathological; *p < 0.05, in a multiple comparison Finner post-hoc test against the proposal

## Discussion

In this work, we sought to successfully classify pulmonary findings in LUS videos. The proposed DL-based framework proved to be flexible, working in both categorical and multi-label problems, and obtaining a promising performance in both. Additionally, the study demonstrated the potential of leveraging unlabeled data in the field of POCUS (besides ultrasound in general), where data availability is not always an issue but its annotation often is.

To effectively handle video data, we opted for the use of 3D CNNs. Among the tested architectures, the selected R2+1D model emerged as particularly suitable (see supplementary material), allowing 3D data processing but requiring fewer parameters, thus mitigating concerns related to overfitting.

In turn, the SSL block had a substantial impact on the final output, even when the total number of (labeled and unlabeled) training samples was kept consistent with the supervised variant (see Section V of the supplementary material). Interestingly, when using the classical pseudo-labeling method ($\tau$_p_ = 0.5 and $\tau$_n_ = 0.5), in which all unlabeled videos are added (and all labels considered reliable), an improvement in F1-scores was observed compared to the supervised counterpart, albeit with the increased training set size. This underscores the potential of these algorithms in such contexts. The introduction of the uncertainty estimation enabled more refined pseudo-label selection and leads to further improvements in the classifier’s performance, corroborating the observations made in [22]. Indeed, a robust model should ideally produce consistent or similar results for the same clip under weak augmentations. Significant variation in outcomes suggests the network’s lack of confidence in the assigned class, prompting a reconsideration of label reliability through uncertainty-based selection. Nonetheless, applying this approach to categorical problems, characterised by fewer labels, did not yield substantial performance improvements (see Table 7). This suggests that it may not be beneficial for problems with lower complexity and requiring fewer data.

Leveraging domain knowledge was also crucial, as evidenced both in terms of the network’s input size and the ensemble modeling. Regarding the former, as demonstrated in supplementary material, using 4-second video clips achieved the best overall performance, which is coherent with physiological knowledge on the average duration of a breathing cycle. Concerning the latter, and consistent with the intuition that models with a higher number of low-level labels are more refined, results from Table 3 demonstrate that ensembles leveraging low-level labels to provide the corresponding high-level labels outperformed models relying solely on those high-level labels, though this improvement was not statistically significant. Conversely, test-time augmentation yielded the worst results, despite still surpassing a single test-time inference.

When comparing both low and multi-output to high-level ensembles, the difference between average F1 and AP scores is minimal and not consistent across metrics. While leveraging a model of just low-level labels to obtain high-level labels is advantageous, relying on models capable of classifying distinct output label sets is presumably more promising. A possible reasoning is that models with distinct label sets focus on distinct data distributions, differing in intra- and inter-class variability, but also class imbalance and label noise. For the low-level labels model ensemble, the proposed approach once again outperformed test-time augmentation, as well as the single inference routine.

However, it is important to note that, although improving the overall outcome, the SSL stage is responsible for causing a calibration issue in the network’s output. This result confirms a known SSL issue, particularly for methods from the proxy-label group: confirmation bias, where incorrect pseudo-labels guide subsequent training iterations and lead to a loop of self-reinforcing errors [31]. For each class, the SSL model’s calibration is farther from optimal when compared to the supervised one. This is particularly noticeable for classes “Less than 3 B-lines” and “3 or more B-lines”, which present the highest expected calibration error (data not shown) and the largest difference between training stages. It is worth noting that these are among the hardest findings to annotate, and likely the classes for which the pseudo-labels inject further noise into the training process. Altogether, this indicates that, although delivering better results than the supervised version, the SSL-derived network is overly confident, which prevents multiple cycles of SSL training.

Regarding the inference routine (Sect. “Video-Level Inference Routine”), the proposed method outperformed both the inference of the entire video and the use of a single video clip (see supplementary material), reducing uncertainty and enhancing robustness.

By observing Tables 4 and 5, a slight decrease in performance is seen between validation and test sets, indicating a potential overfit to the former. However, other factors may also contribute to this decrease, such as varying class imbalance ratios (jeopardising the performance measured for certain low-frequency classes) or higher label noise on the test set. Additional reasons may include the quality of the test set videos or the fact that this set contains fewer videos, increasing the representativeness of each video in the result. Among the classes, considerable differences in performance are observed. Beyond the inherent variability in label difficulty, the clip-level labeling strategy (assigning video-level labels to shorter clips) may have also impacted these results. While this approach works well for relatively static features, such as A-lines, consolidations, or coalescent B-lines, it is less suited to dynamic features, like those relying on counting B-lines. This limitation likely contributed to the lower F1 scores for “Less than 3 B-lines”, as individual B-lines, especially when fewer than three, tend to be transient. Additionally, the presence of a similar artifact, Z-lines (which resemble B-lines but do not extend to the lower sector boundary and must be disregarded), further complicates reliable counting. In contrast, A-lines achieved the highest F1 scores, likely due to their static nature throughout the video and their higher prevalence in the training set. Despite these feature-specific challenges and performance differences, the overall conclusions remain consistent, with the proposed framework surpassing both baseline models. Leveraging the dataset’s inherent hierarchy and employing SSL led to an average improvement of 3.81% and 4.14% in F1-score for mlS1 and mlS4, respectively.

In the categorical tasks (Tables 7 and 8), the proposed SSL pipeline generally outperforms the supervised counterparts, with the exception of Baseline 2 for cLS1. However, the performance gains are smaller than in the multi-label scenario, likely due to the already strong performance of the pipeline after supervised training alone (Baseline 2). This is attributed to the simpler nature of the categorical task and the larger number of samples per class compared to the multi-label setting.

These methods, however, are not without flaws. First, to enhance the performance of the semi-supervised method, novel calibration strategies, especially tailored for multi-label outcomes, would need to be investigated to address the observed limitations. Additionally, this study focused solely on lung parenchymal fields. To broaden its utility in clinical practice, it would also be essential to incorporate fields such as the diaphragm insertion and lung sliding. Furthermore, to facilitate adoption in the clinics, it is imperative for experts to trust its outputs. This could be achieved by resorting to explainability methods, enabling experts to comprehend the reasons for the network’s output (e.g. identifying spatial and/or temporal focal points). These steps represent indispensable next stages to further enhance the proposed framework.

## Conclusion

A novel DL-based framework has been proposed for the automatic interpretation of lung POCUS videos, which employs a (2+1)D ResNet-based network and incorporates a noise-robust approach (through label smoothing regularisation) while also integrating domain knowledge (via model ensembling). Notably, the framework demonstrates flexibility, being applicable to both categorical and multi-label classification objectives depending on the information one desires to retrieve from the LUS video. Furthermore, it leverages both labeled and unlabeled data through a semi-supervised training stage.

The framework’s performance was validated across categorical and multi-label scenarios, showing its adaptability to various tasks and objectives. For instance, the binary categorical model excels in distinguishing between normal-looking lungs and lungs with an indication of underlying pathology, achieving a F1-score of 92.41%. This capability holds promise for rapid patient screening in emergency scenarios, facilitating triage by providing experts with timely insights into urgent cases. Conversely, the more complex multi-label model, with an average F1-score of 70.45% when considering five relevant LUS findings, offers a more comprehensive understanding of pathology progression and extension. Overall, this framework offers potential for accurate computer-assisted LUS interpretation in clinical practice.

## Supplementary Information

Below is the link to the electronic supplementary material.

Supplementary file 1 (pdf 320 KB)

## Data Availability

The dataset utilized in this study is not publicly available due to ethical restrictions. However, it may be available from the corresponding author upon reasonable request, subject to approval from the relevant ethical committees.

## Footnotes

1 https://github.com/uotw/ClipDeidentifier
