# Supplementary material for: Semi-supervised Ensemble Learning for Automatic Interpretation of Lung Ultrasound Videos
Source: J Imaging Inform Med. 2024 Dec 13;38(5):2664–76. doi: 10.1007/s10278-024-01344-y (PMC12572431; doi:10.1007/s10278-024-01344-y)
Supplement: Supplementary file 1 — (pdf 320 KB) [file 10278_2024_1344_MOESM1_ESM.pdf]

Supplemental document for: Semi-supervised ensemble learning for automatic interpretation of lung ultrasound videos

****, ****, ****, ****, ****, ****, ****, ****

I. Data pre-processing

When analysing LUS videos, one aspect that is evident and potentially unfavourable for its pre-processing is the presence of very hypoechogenic regions inside the image's FOV with the same intensity value as those outside it. This characteristic complicates the accurate estimation of the sector scan's mask. A custom pre-processing algorithm was thus created, whose sole input is the video itself.

Figure S1 shows an example frame of the input LUS video.

1) Binary thresholding: First, a thresholding operation was applied to the video to create a raw mask of the LUS FOV. Although the region outside the sector scan appears to be black, its intensities are not always zero, and therefore the threshold limit was set to 2. The resulting binary map is summed across all frames and the result is once again thresholded to identify those locations in which valid information appears at least once during the video (i.e. value in summed map above 0).

Although the resulting mask contains the relevant pixels inside the sector scan, it also contains all non-zero pixels belonging to the accessory information. To filter these unwanted details, a shape- and area-based filter operation was applied through connected components analysis. In simple terms, only non-squared components with an area above 20,000 pixels were maintained (smaller regions represent text and a large squared region represents the bottom menu present in most videos). An example of the filtered mask is shown in white in Figure S2 and Figure S3. At this stage, a bounding box containing all selected pixels is also created.

2) Fitting of the lateral lines and superior/inferior arcs: Based on the binary mask created, one determines the lateral lines and superior/inferior arcs of the sector scan.

To identify the optimal lateral lines (Fig. S2), the most outward pixels of the binary mask, for both left and right sides, must be identified. This is obtained by two search procedures, each searching for the first non-zero pixels (i.e. boundary pixels) from the respective bounding box limit (left or right) to the middle of it. For either side, a regression for the line that best fits the identified set of pixels' positions is calculated.

The same idea is followed for the superior and inferior arcs (Fig. S3). For the superior arc, by running a search procedure on the image columns, one searches for the boundary pixel with the lower y-coordinate on either half of the bounding box. All top boundary pixels located between these two identified positions are considered as probable points of the superior arc. The superior arc is then estimated as the circumference that best fits them. For the inferior concavity, the process is similar, but the search is applied from the bottom over all columns. The inferior arc is then estimated by fitting a circumference to the detected boundary pixels' positions.

3) Line's confidences estimation: To validate if the fitted lines are accurate and can, indeed, be used to create the sector scan mask, a confidence value is calculated for each line.

For the lateral lines, this confidence is defined by two parameters: the number of masked pixels that are either left of the left line or right of the right line; and the angle of the line in relation to the vertical axis. If the number of points outward of the lines is greater than 500 and the angle is higher than 45 degrees, the line cannot be trusted, and its confidence is set to zero. This can occur for just one line or both. In opposition, if the line can be trusted, the confidence takes the value of 1.

For the arcs, to assure a satisfactory fitting, one compares it with the fitting of a line to the same set of pixels. Briefly, one calculates the percentage of inliers (i.e. points whose distance to the closest point in the regressed arc/line is ≤ 2 pixels) when fitting the circumference and the line. If the percentage of inliers for the circumference is higher, then this value is used as confidence. Otherwise, the confidence is set to zero.

4) Probe's virtual origin estimation: At this stage, one has two lateral lines and two arcs identified. Independently of being able to trust them or not, a problem exists with these

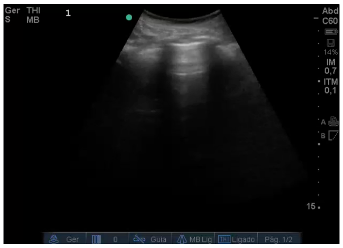

Fig. S1: Example frame of a LUS video before pre-processing.

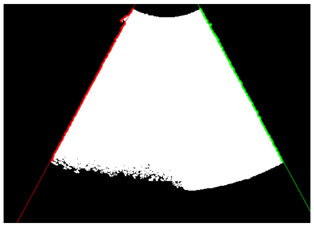

Fig. S2: Fit of the lateral lines.

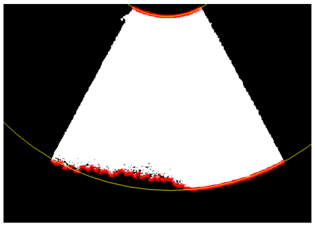

Fig. S3: Fit of the superior and inferior concavities.

calculated regressions: they were all performed independently of each other and, as such, do not have a common vanishing point, which occurs in a real LUS FOV. This is defined as the probe's virtual origin and it is calculated by a weighted average that considers the estimated lines' intersections and their respective confidences. Specifically, five intersections are estimated: the intersection of left and right lines, and the intersections of each lateral line and the vertical line that passes through each arc's centre. The weighted average is obtained by averaging the calculated intersection points' coordinates, weighted by the confidences implied in that intersection, and the arcs' centres, multiplied by their respective confidence.

Once the virtual origin is estimated, to complete the sector scan, both lateral lines and arcs must be adapted to the origin. The virtual origin of the probe will be the mask's centre. This information, along with the opening angle (defined by the lateral lines) and the arcs' radii, must be provided by the algorithm, since they are needed for the data augmentation routine proposed in Section 3.2 of the manuscript.

5) Lines and concavities re-adjustments: To adapt each line, its reliability must be taken into account. If none of the lines can be trusted, the mask will be the bounding box of the initial binary map. Consequently, the probe's virtual origin will be the middle top point of the image, the opening angle will be 180°, the smaller radii will be 0 and the larger will be equal to the image's height. If one trusts the estimated arcs but not the lateral lines, the latter are re-calculated so that the sector scan includes all pixels from the initial binary map. If either one of the lateral lines or even both can be trusted, they will also be recomputed, but in a different manner. A new line is fitted to the set of points originally used for line regression, but adding the constraint that the regressed line must pass through the probe's virtual origin. Since the LUS sector scan is known to be symmetrical, one must further guarantee that these lines are mirrored with respect to the vertical axis. Thus, if both lines were recomputed, their angle with the vertical axis is computed, with the wider angle kept and the other line adjusted. The final angles are used to determine the sector scan's opening angle.

Afterwards, the bounding box is centred in relation to the virtual origin. For the superior arc, the radius is obtained by computing the left lateral line's intersection with the superior bounding box limit, and calculating its distance to the virtual origin. The approach is more straightforward for the inferior concavity since the radius is the distance from the bounding box's bottom limit to the virtual origin.

The identified probe's virtual origin, arcs' radii and lines (Fig. S4) are ultimately used to create a binary mask of the sector scan (Fig. S5), which is used to mask and crop the input video. The coordinates of the virtual origin, the arcs' radii and the opening angle are also stored in a JSON file.

II. Influence of Network Architecture

To evaluate the network's performance alone, one evaluated it in a categorical scenario. Samples were categorised as either "Normal" or "Indicative" following the hierarchy present in Fig. 1. This is the simplest possible problem to create with this dataset so that factors like noisy labels or imbalanced classes influence the least the decision on the chosen architecture.

To validate the selection of the proposed network (a variant of R2+1D), Table S1 compares its performance against five others: the original R2+1D, R3D, C2+1D, C3D [1] and X3D-S [2]. For all architectures, parameters like regularisation weight and learning rate were optimised to guarantee a fair comparison between networks. These architectures were chosen given their similarities with the proposed model and their ability to deal with 3D inputs. Specifically, both R2+1D and R3D were tested as proposed in [1]. In their version, R2+1D is built as to have the same number of total parameters as R3D, which is done by adjusting the number of filters in the network's convolutional layers. For simplification, this operation will be named "boost". In this work, the number of filters is not adapted (no boost), resulting in a network with approximately half of the number of parameters of R3D. Additionally, to verify the advantage of the residual connections, a comparison against traditional CNNs was also considered. Indeed, C3D and C2+1D differ from R3D and R2+1D, respectively, only in the fact that they do not have residual connections. On the other hand, X3D is a highly efficient and scalable architecture designed for video recognition tasks. Its smaller variant (X3D-S) was here employed given the input resolution used.

In short, the chosen network presents the best performance across all metrics. Moreover, ResNet variants seem to outperform non-residual variants, both in 3D or factorised. This aligns with the proven advantages of shortcut connections, avoiding the problem of vanishing and exploding gradients. Another factor for consideration is the number of parameters of each architecture. The proposed R2+1D is one of the networks with fewer parameters (except for X3D-S) but the best performance. Larger networks seem to be overfitting the training data (despite the per-network optimisation of parameters like regularisation weight). This is one of the advantages of the use of factorised blocks compared with 3D ones. Dealing with 3D convolutions automatically increases the number of trainable parameters, which are reduced in the factorised version. This creates a network less prone to overfitting, as demonstrated.

III. Influence of Input Pre-processing

A study on the influence of the network's input, namely the effect of video length at a fixed frame rate and vice-versa, on its performance was conducted. As previously stated,

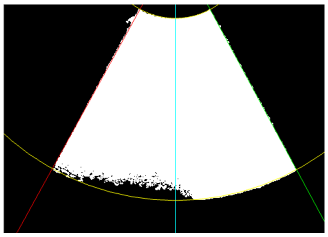

Fig. S4: Example of the final lines and arcs estimated.

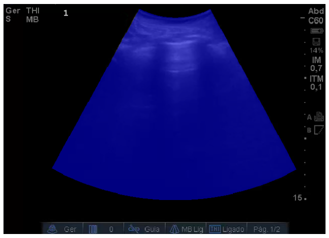

Fig. S5: Example of the final mask (in blue) overlaid on the original image.

a frame rate of 8 Hz and 4 seconds of duration were the chosen parameters. The frame rate was initially defined upon observing videos at different rates and choosing the one that appeared to be the minimum needed for an adequate visualisation of the relevant findings. In its turn, the video length was set to match the average respiratory cycle, which is the minimal length needed to perform a LUS assessment in clinical practice. While a smaller video duration may be insufficient to visualise all relevant findings, a longer video may capture more than just one cycle. Notwithstanding, both options were verified, to choose the most appropriate ones.

Starting with a fixed frame rate of 8 Hz, the length of the video was varied, as observed in Table S2. The results were congruent with the initial expectations, with the best result achieved with 4-second clips. Indeed, successive improvements were seen when increasing the clip length from 2 to 4 seconds, but onward the model's performance deteriorates. For videos with less than 4 seconds, as stated, the justification for the reported results is that most of the clips do not comprehend a complete respiratory cycle. Consequently, there may be information (or even entire findings) missing. With more than 4 seconds, besides possibly covering more than one respiratory cycle, the data variability diminishes given the higher similarity between extracted clips (since their duration gets closer to the length of the original video). With less variability in the training set, the network is more prone to overfitting, which explains the worst performance observed.

Regarding frame rate, Table S3 summarises the results for

TABLE S1: Performance of distinct network architectures

Network	Parameters	BA	MCC	AP	F1-score	
					Normal	Indicative
R2+1D (proposed)	15.4M	**0.9202**	**0.8399**	**0.9702**	**0.9173**	**0.9224**
R2+1D (w/ boost)	33.2M	0.9109	0.8211*	0.9689	0.9067	0.9140
R3D	33.2M	0.9141	0.8273	0.9698	0.9104	0.9167
C2+1D	33.0M	0.9024*	0.8033*	0.9630*	0.8991*	0.9038*
C3D	32.9M	0.9065*	0.8120*	0.9642	0.9029*	0.9086*
X3D-S	3.3M	0.8871*	0.7731*	0.9531*	0.8834*	0.8887*

*p<0.05, in a multiple comparison Finner post-hoc test against the proposed R2+1D.

TABLE S2: Effect of the input's clip duration on the network's performance

Clip duration (seconds)	BA	MCC	AP	F1-score	
				Normal	Indicative
2	0.9063*	0.8112*	0.9677	0.9028*	0.9078*
3	0.9141	0.8275	0.9688	0.9105	0.9169
4	**0.9202**	**0.8399**	**0.9702**	**0.9173**	**0.9224**
5	0.9133	0.8264	0.9666*	0.9096	0.9168
6	0.9010*	0.8011*	0.9613*	0.8963*	0.9046*

*p<0.05, in a multiple comparison Finner post-hoc test against the 4-second clip model.

TABLE S3: Effect of the input's frame rate on the network's performance

Frame rate	BA	MCC	AP	F1-score	
				Normal	Indicative
4	0.9109*	0.8210*	0.9667*	0.9071*	0.9136*
6	0.9127*	0.8247*	0.9691	0.9089*	0.9157
8	**0.9202**	**0.8399**	0.9702	**0.9173**	**0.9224**
10	0.9136*	0.8263*	0.9688	0.9093*	0.9169
12	0.9136*	0.8267*	**0.9707**	0.9098*	0.9168*

*p<0.05, in a multiple comparison Finner post-hoc test against the 8 Hz frame-rate model.

TABLE S4: Comparison between different inference routines

Inference method	BA	MCC	AP	F1-score	
				Normal	Indicative
One-clip inference	0.9105*	0.8206*	0.9638*	0.9073*	0.9129*
Whole-video inference	0.9149*	0.8294*	0.9685	0.9112*	0.9180*
Multi-clip inference (proposed)	**0.9202**	**0.8399**	**0.9702**	**0.9173**	**0.9224**

*p<0.05, in a multiple comparison Finner post-hoc test against the proposed multi-clip inference.

TABLE S5: Comparison between different inference routines

	Training		AP	F1-score	
	First stage	Second stage		Macro	Micro
Supervised learning	D_l	-	**0.6812**	0.6311*	0.6780*
UPS	$0.5D_l$	$0.5D_l$ + $0.5D_{lp}$	0.6508*	0.6386*	0.6811*
	D_l	D_l + D_{up}	0.6786	**0.6638**	**0.7001**

D_l - labeled data, D_u - unlabeled data, D_{lp} and D_{up}- labeled and unlabeled data with pseudo-labels, respectively; * $p < 0.05$, in a multiple comparison Finner post-hoc analysis, against the UPS trained with all (labeled and unlabeled) dataset.

clips with 4 Hz to 12 Hz (and 4 seconds of duration). Frame rates of 4 or 6 Hz led to an inferior performance. This is expected as 4 to 6 Hz may often be insufficient to detect very sudden findings, like an isolated B-line. When increasing the frame rate to a value above 8 Hz, the performance also decreases. By increasing frame rate, while freezing duration, one is increasing the input data depth. However, since the size of the convolutional kernels are kept constant, the associated receptive field is smaller, which means the temporal features extracted concern smaller temporal intervals. Consequently, there is less temporal correlation and more overfitting, which leads to worst results. Overall, the usage a frame rate of 8 Hz reveals to be optimal for the present dataset.

IV. Influence of inference routine

To corroborate the advantages of the proposed video-level inference (average of multiple overlapping video clips), one compared its performance against two others: a whole-video inference and a one-clip inference. For the whole-video prediction, the full video (sampled at 8Hz) is fed to the trained model. For the one-clip prediction, only the first clip of each video is fed to the network. The categorical scenario from the above experiments was once again employed to perform this comparison, whose results are summarised in Table S4.

From the ablation results, it is proved that the average of multiple overlapping clips from the same video is preferred over classifying the video based on a single prediction (of the entire video or one clip). Indeed, a multi-clip inference introduces variability and reduces the model's uncertainty, obtaining more accurate predictions. Between single-prediction inference routines, the use of the full video outperforms the one-clip strategy. These results corroborate the intuition that, by considering the full video, one may identify any finding(s) that appear briefly over the full 6 seconds of the video, which may be missed if one single clip is extracted and analysed.

V. Comparison of supervised and semi-supervised learning with the same amount of training data

Since the supervised learning (SL) approach utilizes only the annotated training data, whereas the semi-supervised counterparts leverage both annotated and additional unlabeled data, it could be argued that this presents an unfair comparison. To address this concern, we conducted an additional experiment in which half of the annotated videos were used for the SL stage, while the remaining half were treated as unlabeled and incorporated into the SSL stage. The total amount of training data in the SSL stage is thus equal to that in the fully supervised baseline.

The results of this experiment are presented in Table S3, alongside the original results for the supervised and UPS approaches. As shown, running the UPS approach with fewer total samples (second row) exhibits lower average precision compared to both the fully supervised and original UPS approaches (first and third rows, respectively). This decline is likely due to model miscalibration arising from the pseudo-labeling process, a challenge also observed in the original UPS model. The issue is further exacerbated here by the smaller amount of labeled data in the initial training stage, which leads to noisier pseudo-labels and reduces the potential performance gains from the additional data in the second stage. Notably, in terms of macro and micro F1-scores, the results indicate that, even with identical training dataset sizes, the UPS model slightly outperforms the supervised model, highlighting the benefits of confidence-aware pseudo-labeling.

References

[1] Tran D, Wang LH, Torresani JR, LeCun Y, Paluri M. A closer look at spatiotemporal convolutions for action recognition. IEEE Conference on Computer Vision and Pattern Recognition. 2018.

[2] Feichtenhofer C. X3d: Expanding architectures for efficient video recognition. Proceedings of the IEEE/CVF conference on computer vision and pattern recognition. 2020; p.203–213.
